# A clinician's guide to the assessment and interpretation of noninferiority trials for novel therapies

**Published:** 2014-05-06

**Authors:** Simon J W Oczkowski

**Affiliations:** Simon J.W. Oczkowski, MD, FRCPC, is an internal medicine specialist and critical care fellow training in the Division of Critical Care at McMaster University, Hamilton, Ontario.

## Abstract

A noninferiority trial is designed to demonstrate that an experimental therapy is not worse than an active control. Although noninferiority trials are superficially similar to conventional superiority trials, there are fundamental differences. In particular, aspects of a study that make the therapies appear more similar than they actually are can falsely bias the study toward demonstrating noninferiority. This has important implications for methodologic techniques such as blinding and statistical analysis based on the intention-to-treat principle. When applying the results of noninferiority trials, clinicians should be judicious in determining whether the degree of noninferiority demonstrated is clinically acceptable and whether the ancillary benefits of the treatment justify its use.

Most clinicians have become familiar with interpreting and applying the results of randomized controlled trials (RCTs) designed to assess whether an experimental therapy is superior to placebo, known as "superiority trials." In contrast, many are less familiar with RCTs designed to demonstrate that the efficacy of an experimental therapy is not significantly inferior to that of a previously proven therapy (called an "active control"). RCTs with such a design are called "noninferiority trials." Noninferiority trials should not be confused with superiority trials that have statistically nonsignificant outcomes, although some study authors do try to put such a positive "spin" on negative superiority trials.^1^

Although noninferiority trials are superficially similar in design to superiority trials, clinicians should be aware of several important ways in which these 2 types of trials differ, so that they can properly assess the validity, significance, and applicability of trial results.^2^ Contributing to the confusion is the fact that many noninferiority trials are poorly reported or do not meet current guidelines designed to reduce the chance of bias.^3^,^4^ This article has been written to help clinicians understand the rationale, design, assessment, and application of noninferiority trials.

## What is a noninferiority trial, and why would such a trial be used?

The most common reason that investigators choose to conduct an noninferiority trial, rather than a conventional superiority trial, is because the therapeutic strategies may have characteristics beyond their overall treatment effect that make them attractive to clinicians and patients, for example, oral anticoagulants that do not require monitoring. In addition, the great efficacy of some current treatments means that new therapies compared against them are likely to have relatively small effects and would require powerful trials with prohibitively large sample sizes in order to demonstrate statistical superiority.^5^ Given these considerations, the number of published noninferiority trials might be expected to increase over time, as has indeed been observed over the past decade.^3^

A brief review of the basic principle behind the design of conventional superiority trials will be helpful in understanding the fundamentally different structure and purpose of a noninferiority trial, despite superficial similarities. The basic principle underlying any clinical trial is the null hypothesis (*H_0_*). In superiority trials, the null hypothesis is most commonly presented as follows:

*H_0_*: For the outcome of interest, treatment x is no more effective than treatment y (or placebo)

The alternative hypothesis (*H_1_*) is typically presented as follows:

*H_1_*: For the outcome of interest, treatment x is more effective than treatment y (or placebo)

In conducting a conventional superiority trial, the aim is to disprove the null hypothesis and show that the 2 treatments differ in effect.

For noninferiority trials, however, the null and alternative hypotheses are quite different:

*H_0_*: For the outcome of interest, treatment x is worse than treatment y by more than ƒ

*H_1_*: For the outcome of interest, treatment x is not worse than treatment y by more than ƒ,

where f is the noninferiority margin.

For completeness, a third type of trial, the equivalence trial, should also be mentioned, although it is rarely used in clinical medicine. Such a trial would have the following null hypothesis:

*H_0_*: For the outcome of interest, treatment x is not worse, nor is it better, than treatment y by more than ƒ

**Figure 1:**
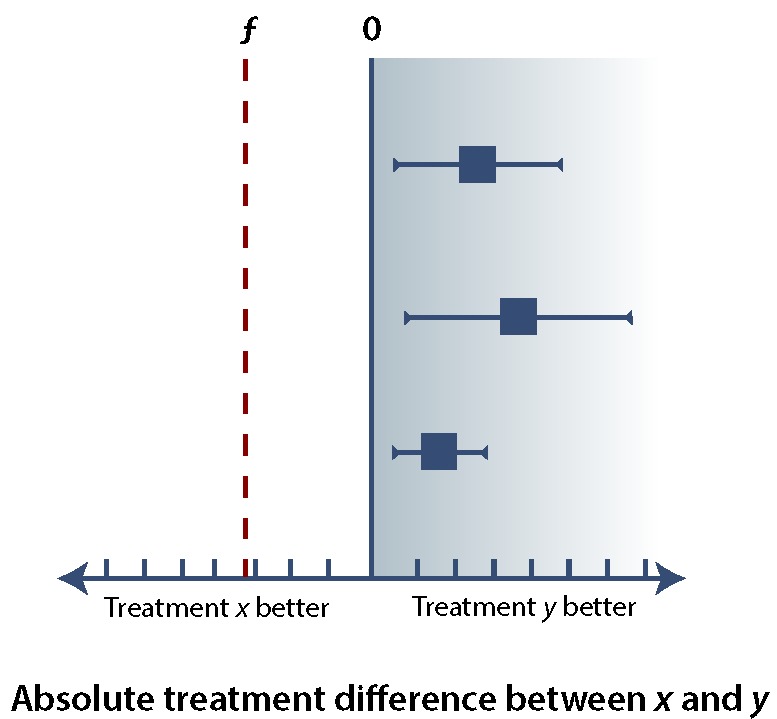
The results of 3 hypothetical trials comparing treatment y with treatment x, illustrating the concept of superiority. In all 3 cases, the lower bound of the 95% confidence interval (CI) is both greater than the noninferiority margin, ƒ, and greater than 0 (blue shaded area). Together, these results indicate the superiority of treatment *y*. As a general principle, for any additional studies, the lower bound of the 95% CI falling within the blue shaded area would indicate superiority of treatment y.

**Figure 2:**
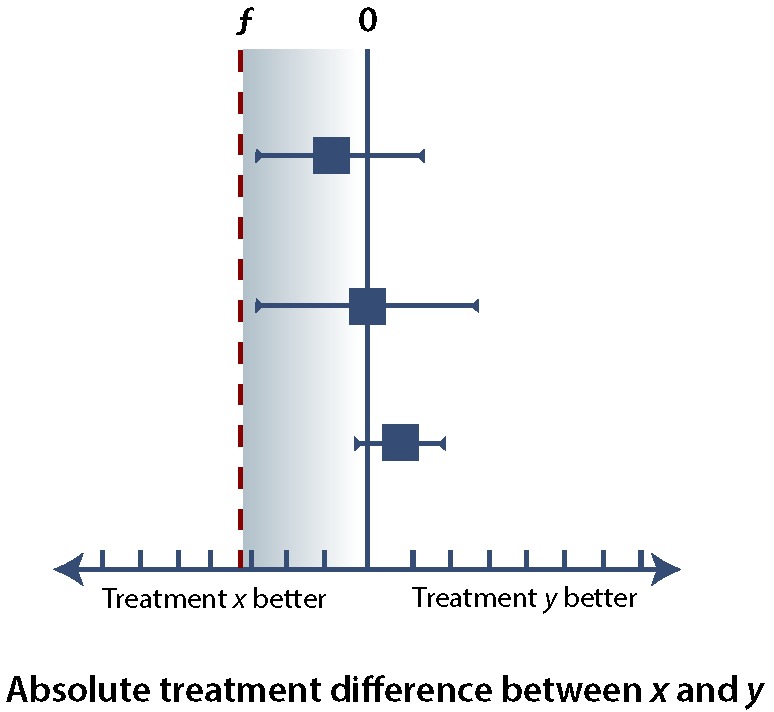
The results of 3 hypothetical trials comparing treatment *y* with treatment *x*, illustrating the concept of noninferiority. In all 3 cases, the lower bound of the 95% confidence interval (CI) is less than 0; as such, all of these trials fail to demonstrate superiority of treatment *y* over treatment *x*. However, the lower bound of each 95% CI lies above the noninferiority margin, ƒ (blue shaded area); as such, all of these trials demonstrate noninferiority of treatment *y* with respect to treatment *x*. As a general principle, for any additional studies, the lower bound of the 95% CI falling in the blue shaded area would indicate noninferiority of treatment *y* with respect to treatment *x*.

**Figure 3:**
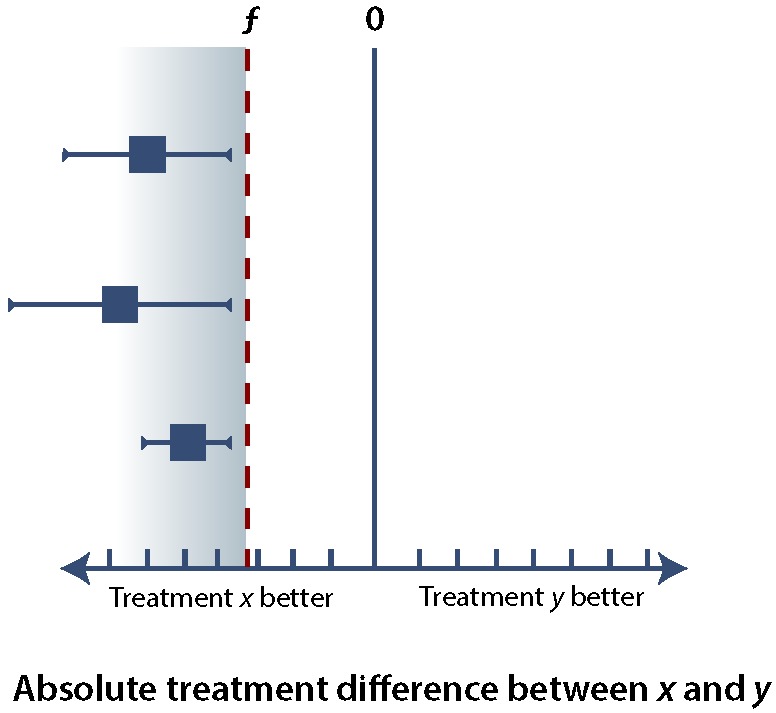
The results of 3 hypothetical trials comparing treatment *y* with treatment *x*, illustrating the concept of inferiority. In all 3 cases, both the lower bound and the upper bound of the 95% confidence interval (CI) are below the noninferiority margin, ƒ (blue shaded area); as such, all of these trials demonstrate inferiority of treatment *y* with respect to treatment *x*. As a general principle, for any additional studies, the upper bound of the 95% CI falling in the blue shaded area would indicate inferiority of treatment *y* with respect to treatment *x*.

**Figure 4:**
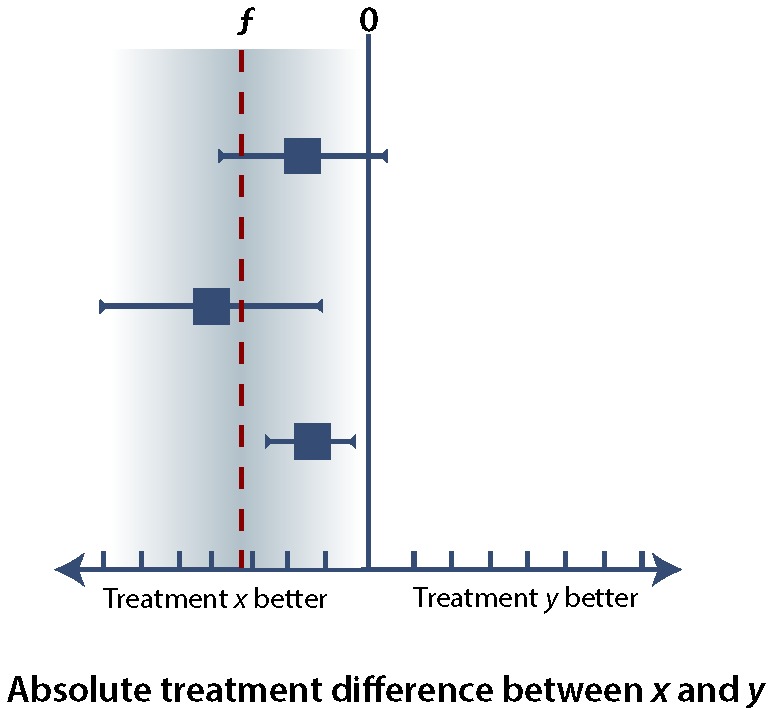
The results of 3 hypothetical trials comparing treatment y with treatment *x*, illustrating potentially indeterminate results. In the topmost trial, the lower bound of the 95% confidence interval (CI) falls below the noninferiority margin, ƒ; as such, even though noninferiority has not been demonstrated, it is still possible that treatment *y* is noninferior, because ƒ is included within the 95% CI. In addition, because the upper bound of the 95% CI is greater than 0, it is impossible to say whether treatment *y* is inferior to treatment *x*. In the middle trial, the upper bound of the 95% CI is less than 0, which indicates that treatment y is inferior to treatment *x*; however, it is possible that the effects of treatment *y* would fall within the noninferiority margin, since ƒ is included within the 95% CI. In the bottom trial, the 95% CI falls within the space between ƒ and 0. In this case, treatment *y* has been demonstrated to be inferior to treatment *x*, and at the same time, it meets the criterion for noninferiority compared to treatment *x*. This situation reinforces the importance of selecting an appropriate noninferiority margin, since, in theory, a treatment that is definitely worse than the active control but no more so than ƒ can be technically "noninferior." Such a result requires a very narrow, precise 95% CI and is unlikely to occur except in studies with very large sample sizes.

In any of these types of trials, it is important to minimize the error of falsely rejecting or accepting *H_0_* (see Box 1). Ensuring an α error rate of 5% or less for superiority, noninferiority, or equivalence trials requires that the (1 – α) confidence interval (CI), usually a 95% CI, falls within specific margins. If we consider the difference in effect between treatment *x* and treatment *y* (i.e., the treatment effect of *x* minus the treatment effect of *y*), superiority implies that the lower bound of the 95% CI is greater than 0 (Figure 1), whereas noninferiority allows the 95% CI to extend below 0, so long as it remains above the noninferiority margin, ƒ (Figure 2). If the upper bound of the 95% CI falls below the noninferiority margin, ƒ, then the trial has failed to demonstrate noninferiority and has in fact demonstrated inferiority (Figure 3). If the 95% CI crosses ƒ, then the study result is indeterminate. There are several possible scenarios in which the result may be indeterminate (Figure 4). In the uncommon case of an equivalence trial, the upper and lower boundaries of the 95% CI must fall between ƒ and –ƒ to successfully demonstrate equivalence (not shown).

As with superiority trials, the adoption of an α error rate of 5% in noninferiority trials is by convention. Likewise, the selection of the noninferiority margin is at the researcher's discretion, and recommendations on how it should be determined, and therefore how much worse a new treatment can be to be considered "noninferior," also vary widely.^6^ Ideally, the noninferiority margin should be the smallest difference in efficacy between 2 treatments that would be considered clinically relevant to a physician and his or her patients. In practice, most researchers select a noninferiority margin to retain 80% to 85% of the clinical effect of the active control.^7^ In addition, the clinical efficacy of the active control treatment must be well established. An example of a hypothetical trial comparing a new antibiotic with meropenem for treatment of hospital-acquired pneumonia according to both superiority and noninferiority criteria is shown in Table 1.

**Table 1:** Design of a hypothetical trial comparing a new antibiotic with meropenem as a superiority trial or as a noninferiority trial, with a noninferiority margin of 2% for the outcome of clinical cure

Element of study	Superiority trial	Noninferiority trial
Null hypothesis (*H_0_*)	The new antibiotic does not have a higher cure rate than meropenem	The new antibiotic has a cure rate more than 2% lower than that of meropenem
Alternative hypothesis (*H_1_*)	The new antibiotic does have a higher cure rate than meropenem	The new antibiotic has a cure rate no more than 2% lower than that of meropenem
Difference between treatments required to disprove null hypothesis (*H_0_*)	The lower bound of the 95% confidence interval must be greater than 0	The lower bound of the 95% confidence interval must not be lower than –2%
Factors that improve the likelihood of disproving null hypothesis (*H_0_*)	Factors that result in greater differences between study groups	Factors that result in smaller differences between study groups

Box 1 **Types of errors in hypothesis testing**

Two types of errors can be made in drawing conclusions from a hypothesis test:

- Type 1 (α) error: the probability of rejecting the null hypothesis *H_0_* if it is true
- Type 2 (β) error: the probability of accepting the null hypothesis *H_0_* if it is false

The type 1 error represents the significance of a test result if a difference between the 2 treatments is demonstrated. Most clinicians are familiar with the similar concept of *p* value. The determination of what constitutes a "significant" *p* value for a particular outcome is based on the type 1 (α) error rate that the researcher is willing to accept. Usually, values of *p* ≤ 0.05 are deemed to be significant, which means that the chance of a test having a result of this magnitude or greater by chance alone is 5% or less. This gives the test an α error rate of 5% (meaning the researcher will falsely reject the null hypothesis 5% of the time).

Conversely, type 2 error is more commonly understood as 1 – β, the statistical power of a test. The more powerful the test, the less likely the researcher is to falsely accept the null hypothesis (and thus, to believe there is no difference between 2 treatments when in fact a difference exists).

Although subtle, the differences in null hypotheses (*H_0_*) between superiority and noninferiority trials have profound implications. For a superiority trial to be successful in disproving *H_0_*, it should maximally demonstrate the difference in efficacy between the experimental therapy and the active control. Thus, any methodologic issues that minimize differences between the 2 treatments will weaken the evidence for the experimental therapy. By contrast, for a noninferiority trial to be successful in disproving *H_0_*, it should maximally demonstrate similarity in the effects between 2 treatments, and any methodologic issues that minimize differences between the 2 treatments will falsely strengthen the evidence for the experimental therapy. As such, the clinician must modify several considerations when appraising a noninferiority trial. Here, I have adapted the 3-step assessment used by Guyatt et al.^8^,^9^ (see Box 2).

Box 2 **Suggested approach to assessing noninferiority trials**

Are the trial results valid?

- Were participants in the trial appropriately randomized, with concealment of the allocation?
- Were all relevant groups (participants, caregivers, data collectors, outcome adjudicators, statisticians) blinded to treatment allocation?
- Were relevant groups blinded to study design (e.g., noninferiority trial v. superiority trial), or did the study also have prespecified superiority criteria of which groups were aware?
- Was follow-up complete?
- Are the results consistent between per-protocol and intentionto- treat analyses?

What are the trial results?

- What were the 95% confidence intervals for primary outcomes?
  - Did the trial meet the prespecified noninferiority margin?
- Were subgroup analyses prespecified?
- Did the trial assess the superiority of the new treatment in terms other than the primary outcomes (e.g., convenience, cost, patient satisfaction, quality of life)?

How can I apply the trial results to my patient?

- Is my patient similar to those studied in the noninferiority trial?
- Is my patient similar to those in previous studies that demonstrated the efficacy of the control treatment?
- Is the potential risk in loss of efficacy of the experimental treatment outweighed by its ancillary benefits?

## How should noninferiority trials be interpreted?

### Are the trial results valid?

The validity of the study results refers to whether the results are biased (i.e., systematically deviating from the truth). In a noninferiority trial, the usual considerations with regard to appropriate randomization and concealed allocation of study participants apply, as do concerns about loss to follow-up. However, there are 2 specific areas where a clinician should exercise special caution when interpreting the results of a noninferiority trial: blinding and method of analysis.

### Blinding

Blinding of study participants, clinicians, outcome assessors, data collectors, and statisticians whenever possible can avoid bias caused by the placebo effect and by co-intervention; however, in noninferiority trials, the extent of reduction of bias achievable by blinding may be limited. For example, say a researcher adjudicating an outcome has, consciously or unconsciously, a bias toward the new treatment *x*, which is actually inferior to the active control. When assessing each study participant for a given outcome, the researcher is blinded to treatment allocation. In a superiority trial, the researcher clearly cannot bias results in favour of treatment *x* as long as the blinding is maintained. However, in a noninferiority trial, even a blinded researcher can bias results by reporting similar outcomes in all patients irrespective of treatment group. Doing so would make treatment *x* appear to be identical with the active control and, hence, falsely noninferior.

There are ways by which blinding can be improved in noninferiority trials. First, all study participants and most study investigators could be blinded as to the study design (e.g., superiority trial v. noninferiority trial). If researchers are unaware whether trial success depends on showing a difference or a similarity between the 2 interventions, they will be unable to bias the results. A second, perhaps more practical approach would be to avoid dedicated noninferiority trials altogether, and design superiority trials with prespecified noninferiority margins. This approach would restore the efficacy of blinding as used in superiority trials, while still allowing investigators to use a noninferiority margin in cases where noninferiority is a sufficient outcome. In summary, it is important for clinicians to know that, unless trial methodology was blinded or the primary study design was a superiority trial with prespecified noninferiority margins, blinding in the context of a noninferiority trial may not result in as significant a reduction of bias as would be expected in a superiority trial.

### Method of analysis

It is generally taught that intentionto- treat analysis is preferable for clinical trials, because it produces a more conservative estimate of effect than a per-protocol analysis and thus reduces the risk of type 1 (α) error. Patients who fail to adhere to the study protocol are thought, on average, to have a worse prognosis than those who manage to remain adherent. Analyzing trial participants in the groups to which they were originally assigned, irrespective of the treatments received, minimizes bias introduced by the drop-out of patients with poor prognosis, and thus intention-to-treat analysis makes the 2 treatment groups appear more similar. In noninferiority trials, however, this approach can actually increase the possibility of type 1 (α) error, as a potentially inferior therapy can falsely appear to have similar efficacy to a proven treatment. Hence, a per-protocol analysis, with its tendency to exaggerate the differences between treatment groups, will actually yield a more conservative estimate in the setting of a noninferiority trial. A noninferiority trial that has similar results when analyzed using per-protocol and intention-to-treat methods is therefore more likely to represent a truly significant result than one with results that are inconsistent between the 2 types of analyses.

### What are the trial results?

The results of a noninferiority trial should clearly state the 95% CIs for the primary outcomes of interest and the results for any prespecified subgroup analyses. Just as important, given that one of the rationales for conducting a noninferiority rather than a superiority trial is that the treatment of interest may have an inherently desirable quality (e.g., lower dosing frequency, less frequent monitoring, or lower cost), results pertaining to this outcome should also be available and ideally should be important to patients. Because there is a theoretical possibility that the "noninferior" treatment will be worse (up to the prespecified margin of ƒ), there should also be evidence that its use carries another benefit of interest to patients.

For example, say the new antibiotic from the example in Table 1 allows for once-daily dosing, rather than the thrice-daily dosing used for meropenem, but is unlikely to have greater efficacy, since both antibiotics have a high likelihood of resulting in clinical cure. A study investigator may decide to conduct a noninferiority trial, because if such a trial is successful, clinicians could then prescribe the new drug with confidence, and patients could enjoy the benefits of its convenient dosing schedule. However, if this is the rationale for conducting the noninferiority study, the researchers should also collect and publish data relating to patient-reported convenience. If patients do not find the daily dosing to be significantly more convenient, it is difficult to justify prescribing the new drug given that it may still be inferior to meropenem by as much as the prespecified margin of ƒ. It is this trade-off between the benefits of the new treatment and the probability that it may not be as effective as the active control, by as much as ƒ, that clinicians must consider when interpreting noninferiority trials. If the study results do not also evaluate the study treatment's potential ancillary benefits over the active control, such a determination is difficult to make.

### Can I apply the trial results to my patient?

As with superiority trials, it is important to consider whether there is any reason to believe that the patient to whom the trial results are to be applied is dissimilar from those in the study, in which case the study results may not be relevant. Beyond this concern, the most important issue to consider when applying the results of a noninferiority trial is not whether the lower bound of the 95% CI lies above the noninferiority margin selected by the researchers (which would indicate statistical noninferiority), but whether this lower bound indicates an efficacy for the outcome that would be acceptable to a patient (i.e., clinical noninferiority).^10^ For an outcome such as death, a noninferiority margin of –2.5% (and hence a possible increased mortality risk of up to 2.5% with use of the new treatment) may be statistically noninferior, but may still be clinically inferior, especially given that the absolute risk increase for mortality gives a number needed to harm of 40. Conversely, for a minor outcome (such as pruritus) a noninferiority margin of –2.5% may be reasonable. As mentioned above, the clinician should also consider the magnitude of other ancillary benefits of the treatment (e.g., dosing convenience), and the relative importance of these outcomes to patients should be carefully weighed.

For example, in the RACE II trial, which compared strict versus lenient rate control in atrial fibrillation, the researchers selected a noninferiority margin of 10%, meaning that lenient rate control compared with strict rate control could have an absolute 10% increase with regard to the primary outcome, a composite of death from cardiovascular causes, hospital admission for heart failure, stroke, systemic embolism, bleeding, arrhythmic events, and implantation of a pacemaker. The study demonstrated an absolute difference of –2.0% (90% CI –7.6% to 3.5%), which satisfied the noninferiority margin.^11^ However, the 90% CI extended to a possible increase in the composite outcome of up to 3.5%. The question of whether this possible increase in the rate of the composite outcome, which includes important elements such as cardiac death and stroke, would be outweighed by the convenience of lenient rate control, is debatable. The investigators published data about frequency of adverse reactions and hospital admissions, but they did not publish any data relating to whether lenient rate control was easier or more convenient for patients— information that would help patients and clinicians decide whether lenient rate control was an acceptable therapeutic strategy.

## Conclusion

There are several reasons why a noninferiority trial may be a preferred study design for a therapeutic intervention, and there are many ways in which the design of a noninferiority trial differs from that of a superiority trial. Clinicians should be aware of these differences when interpreting the results of noninferiority trials, to ensure that the results of the trial are appropriately applied to their patients. Major areas where the 2 types of trials differ include reduced effectiveness of blinding (in the absence of blinding participants to methodology, or the use of prespecified superiority criteria in addition to a noninferiority margin) with noninferiority trials; the more conservative estimate by per-protocol rather than intention-to-treat analysis; the importance of assessing the superiority of nonprimary outcomes of interest to patients; and the critical importance of deciding whether the study provides clinically significant information about patient care, as opposed to statistically significant information about an arbitrary noninferiority margin.
